# A qPCR-based approach targeting the microbial gene marker *nanA* of mucin-degrading *Akkermansia* in Parkinson's disease

**DOI:** 10.1177/1877718X261462342

**Published:** 2026-08-10

**Authors:** Yasuaki Mizutani, Tadashi Fujii, Yasuhiro Maeda, Kohei Funasaka, Eizaburo Ohno, Yoshiki Hirooka, Hirohisa Watanabe, Takumi Tochio

**Affiliations:** 1 Department of Neurology, 12695 Fujita Health University School of Medicine, Toyoake, Aichi, Japan; 2 Department of Gastroenterology and Hepatology, 12695 Fujita Health University School of Medicine, Toyoake, Japan; 3 Department of Medical Research on Prebiotics and Probiotics, 12695 Fujita Health University, Toyoake, Japan; 4 BIOSIS Lab. Co., Ltd, Toyoake, Japan; 5 Open Facility Center, 12695 Fujita Health University, Toyoake, Aichi, Japan

**Keywords:** Parkinson's disease, microbiota alteration, quantitative PCR, *Akkermansia*, *Akkermansia nanA* gene

## Abstract

**Background:**

Parkinson's disease (PD) is a multifactorial neurodegenerative disorder increasingly linked to gut microbiota alterations. However, despite advances in fecal microbiota profiling as a non-invasive approach to disease risk assessment, its clinical utility remains limited by a lack of functionally relevant microbial biomarkers.

**Objective:**

This cross-sectional study aimed to identify a microbial gene marker reflecting metabolic potential associated with both the presence and severity of PD.

**Methods:**

Fecal samples from patients with PD (*n* = 59) and healthy controls (*n* = 65) were analyzed by 16S rRNA sequencing to characterize taxonomic profiles. Quantitative PCR (qPCR) targeted the consensus sequence of the mucin-degrading *nanA* gene (*nan_Akk_*), a highly conserved within *Akkermansia nan* gene clusters. Differences in taxonomic composition and *nan_Akk_* abundance were examined, and correlations with clinical severity scores evaluated in the PD group.

**Results:**

Patients with PD showed reduced abundance of short-chain fatty acid-producing taxa (*Faecalibacterium*, *Blautia*, and *Anaerostipes*) and increased levels of *Akkermansia*. *Akkermansia* abundance correlated positively with motor severity, including Hoehn–Yahr stage. Moreover, *nan_Akk_* levels also correlated positively with Hoehn–Yahr stage and were significantly elevated in PD patients compared with controls. Levels in the stage 4–5 group exceeded those in the stage 1–3 group (*P* = 0.0202), indicating a stage-related increase in mucin-degrading *nan_Akk_* abundance.

**Conclusions:**

We have identified *nan_Akk_* as a microbial gene marker associated with both the presence and severity of PD. Our qPCR-based quantification shows potential as a non-invasive biomarker for disease stratification.

## Plain language summary

A stool test measuring a mucin-degrading Akkermansia nanA gene may help indicate Parkinson’s disease severity.

Parkinson's disease affects not only the brain but also other parts of the body, including the gut. Many studies have shown that people with Parkinson's disease have changes in their gut bacteria. However, most available tests describe which bacteria are present rather than what functions they have. This study aimed to identify a simple stool-based marker that reflects the functional potential of gut bacteria related to Parkinson's disease.

We focused on Akkermansia, a gut bacterium known for its ability to break down the intestinal mucus layer. This bacterium can increase intestinal permeability under certain conditions, and it also contributes to mucus renewal and immune balance in healthy states. We focused on a gene called nanA, which enables Akkermansia to use sialic acid from the gut's mucus layer as a nutrient source, and quantified this gene (referred to as “nanAkk”) in stool samples from people with Parkinson's disease and healthy individuals using a PCR-based method.

Levels of nanAkk were higher in the Parkinson's disease group than in healthy controls. The levels of nanAkk also increased stepwise across Hoehn-Yahr stages, meaning that people in more advanced stages had higher gene levels. This relationship with Hoehn-Yahr stage remained even after taking age, sex, and medication dose into account. In contrast, nanAkk did not show clear associations with blood-based metabolic or inflammatory markers.

These findings suggest that measuring the Akkermansia nanA gene in stool could serve as a non-invasive marker of microbial features associated with both the presence and severity of Parkinson's disease. This PCR-based approach may help link gut microbial function to clinical progression and complement sequencing-based microbiome analyses.

## Introduction

Parkinson's disease (PD) encompasses heterogeneous neurodegenerative and systemic processes that extend beyond the central nervous system.^1^ Increasing evidence suggests that gut microbiota alterations may contribute to both the pathogenesis and progression of PD via the gut–brain axis. Patients with PD consistently show altered microbial composition, with depletion of short-chain fatty acid (SCFA)-producing taxa such as *Faecalibacterium*, *Blautia*, *Fusicatenibacter*, and *Anaerostipes*, and enrichment of pro-inflammatory bacteria, including *Akkermansia muciniphila* (*A. muciniphila*) and *Escherichia coli*.^2–4^

Among these, *A. muciniphila* has attracted considerable attention due to its ability to degrade the mucus layer and its association with increased gut permeability. Based on prior studies, it has been hypothesized that these effects may promote systemic inflammation and α-synuclein aggregation in the enteric nervous system, which are considered key early events in PD pathogenesis.^5,6^ However, the role of *A. muciniphila* appears complex: while its mucin-degrading activity can disrupt the mucus barrier under pathological conditions, it also contributes to mucosal renewal and immune homeostasis in health.^7,8^ This functional duality highlights the need for a more precise assessment of *Akkermansia* activity based on its metabolic potential. However, a metabolically relevant and quantitative PCR (qPCR)-ready microbial gene marker reflecting PD severity has not yet been demonstrated.

In our previous work, we proposed that microbial gene markers, particularly those with metabolic relevance, offer unique advantages as biomarkers, since their abundance reflects the metabolic potential among microbial taxa associated with disease processes. We have also shown that such markers can be quantified rapidly and economically using a qPCR platform, thereby offering a practical alternative to next-generation sequencing (NGS) for potential use in clinical settings.^9,10^

One such functionally relevant gene marker is *nanA*, which is involved in the utilization of host-derived sialic acid from the gut mucus layer by gut microbes.^11^ Because this metabolic activity relates to microbial interactions with the intestinal barrier, *nanA* abundance may reflect disease-associated microbial functional potential. Homologous *nan* clusters have been identified in various mucin-degrading gut bacteria, such as *Mediterraneibacter gnavus* (formerly *Ruminococcus gnavus*) and members of the Lachnospiraceae family, indicating that the capacity for sialic acid metabolism is broadly conserved among commensal microbes.

Consistent with previous reports in other mucin-degrading gut bacteria, *A. muciniphila* has also been shown to possess a *nan* gene cluster involved in sialic acid catabolism.^12,13^ As in other mucin-degrading bacteria, *nanA* in *A. muciniphila* represents a gene marker reflecting the functional potential for mucin degradation in the gut microbiota.

Beyond mucin degradation, microbial metabolic activity can affect host energy and redox balance through the production of metabolites such as SCFA and tricarboxylic acid (TCA) cycle intermediates.^14,15^ Disturbances in these pathways, along with altered adenosine phosphates (e.g., ATP, ADP) and nicotinamide cofactors (e.g., NAD^+^, NADP^+^), have been implicated in PD pathophysiology.^16–18^ We also assessed the neutrophil-to-lymphocyte ratio (NLR) as a readily accessible indicator of systemic inflammation, given its reported association with PD severity and immune dysregulation.^19^ Integrating these metabolic and inflammatory indices with microbial data may clarify how gut microbial metabolic potential contribute to systemic homeostasis and disease progression in PD.

This study therefore focuses on the abundance of microbial genes linked to metabolic potential, aiming to advance the development of non-invasive, rapid, and cost-effective strategies for detecting and monitoring PD-related microbial alterations. In addition, we have examined their potential associations with systemic energy metabolism and inflammatory markers. Taken together, such microbial gene markers may hold particular promise for identifying high-risk individuals, facilitating longitudinal surveillance, and enabling early-stage PD screening in aging populations.

## Methods

### Patients and fecal sample collection

This cross-sectional study enrolled 59 consecutive patients with PD, who visited Fujita Health University Hospital (Toyoake, Japan) as either inpatients or outpatients between January 2023 and March 2025. All patients met the clinical diagnostic criteria for PD established by the Movement Disorder Society.^20^ Fecal samples were collected using a FS-0015 collection kit (Techno Suruga Laboratory, Shizuoka, Japan).

### Healthy control individuals and fecal sample collection

Fecal samples were obtained from healthy individuals (≥50 years, *n* = 65; mean age: 67 years; 49.2% male), and had been analyzed previously in our earlier study.^21^ Fecal sample collection was performed in the same manner as described above.

### Clinical data collection

Motor and non-motor symptoms of PD were comprehensively assessed using standardized clinical scales. The Japanese version of the Movement Disorder Society–Unified Parkinson's Disease Rating Scale (MDS–UPDRS) was used to determine overall disease severity,^22^ with the Hoehn–Yahr stage used to classify motor severity.^23^ Cognitive function was assessed using multiple tools, including the Mini-Mental State Examination (MMSE),^24^ Addenbrooke's Cognitive Examination-Revised (ACE-R),^25^ Frontal Assessment Battery (FAB),^26^ and the Japanese version of the Montreal Cognitive Assessment (MoCA-J).^27^ Additional assessments were performed for quality of life (Parkinson's Disease Questionnaire-39, PDQ-39),^28^ depression (Geriatric Depression Scale-15, GDS-15),^29^ olfaction (Odor Stick Identification Test for Japanese, OSIT-J),^30^ autonomic function (Scales for Outcomes in Parkinson's Disease-Autonomic, SCOPA-AUT),^31^ daytime sleepiness (Epworth Sleepiness Scale, ESS),^28^ and rapid eye movement (REM) sleep behavior (REM Sleep Behavior Disorder Screening Questionnaire, RBDSQ-J).^32^ The Constipation Severity Score was calculated as the sum of items 5 and 6 of the SCOPA-AUT; this provided a continuous indicator of constipation severity. The levodopa equivalent daily dose (LEDD) was calculated according to the most recent conversion formula.^33^ All assessments were conducted during the “on” state. The interval between these clinical assessments and stool collection was 7.2 ± 16.9 days (mean ± SD).

### 16S rRNA gene and *Akkermansia nanA* gene analyses

Fecal DNA was extracted using a commercial kit and subjected to 16S rRNA gene NGS targeting the V3–V4 region, following previously established protocols for the MiSeq platform.^9^ Taxonomic and functional analyses were conducted using the EzBioCloud 16S-based MTP pipeline (ChunLab Inc., Seoul, Korea). After sequence processing and taxonomic assignment, a total of 667 genera and 1800 species were identified across both the control and PD groups, which were subsequently included in the downstream statistical analysis. Alpha diversity indices (Chao1, Shannon) and beta diversity (generalized UniFrac distance) were calculated, and group differences compared using Wilcoxon rank sum tests and permutational multivariate analysis of variance (PERMANOVA). Differential taxa and functional pathways were identified by linear discriminant analysis (LDA) effect size (LEfSe) with thresholds of *P* < 0.05 and |LDA| > 3.7. To quantify the *nanA* gene encoding N-acetylneuraminate lyase (which is specific to *Akkermansia* spp.), genus-conserved regions were identified and a primer set designed based on homologous sequences from representative *Akkermansia* strains (Supplemental Figure 1). qPCR was used to determine the abundance of the *Akkermansia nanA* gene (*nan_Akk_*), normalized to total bacterial 16S rRNA levels. In selected samples, *nan_Akk_* amplicons were further subjected to MiSeq-based sequencing for validation of primer specificity and strain-level variation. Detailed protocols for primer design, qPCR conditions, and NGS processing are provided in Supplemental Methods 1.

### Blood sample collection

After a fasting period of more than 6 h, venous blood was collected from patients with PD using EDTA 2Na-containing tubes. The interval between blood and stool sample collection was 2.7 ± 2.2 days (mean ± SD). Whole-blood purine nucleotide/cofactor analyses were available for 51 participants, and plasma energy metabolism-associated metabolite analyses were available from 50 participants.

### Measurement of purine nucleotides and related cofactors in whole blood

Whole-blood concentrations of adenosine monophosphate (AMP), adenosine diphosphate (ADP), adenosine triphosphate (ATP), nicotinamide adenine dinucleotide (NAD), nicotinamide adenine dinucleotide phosphate (NADP), and nicotinamide mononucleotide (NMN) were measured by liquid chromatography–tandem mass spectrometry according to previously published methods.^34^ The analytical method is described in detail in Supplemental Methods 2.

### Measurement of energy metabolism-associated plasma metabolites

Plasma concentrations of SCFAs, medium-chain fatty acids, and TCA-related organic acids were analyzed using ultra-high-performance liquid chromatography-tandem mass spectrometry. The analytical method is described in detail in Supplemental Methods 3.

### Systemic inflammatory index

The NLR was calculated as a systemic inflammatory index from peripheral blood cell counts obtained on the same date as blood collection.

### Statistical analysis

All statistical analyses were performed using GraphPad Prism version 10.3.1 (GraphPad Software, San Diego, CA, USA) and JMP software, version Pro 18 (SAS Institute, Cary, NC, USA). A *P*-value <0.05 was considered statistically significant. Comparisons of age between groups were performed using the Mann–Whitney *U* test. Differences in sex distribution were examined using the Chi-square test. Correlations between microbial abundance or qPCR results and clinical scores were assessed using Spearman's rank correlation analysis. Group comparisons of qPCR-based gene quantification were conducted using the Kruskal–Wallis test, followed by the two-stage linear step-up procedure of Benjamini, Krieger, and Yekutieli to correct for multiple testing. To correct for multiple comparisons, a false discovery rate (FDR) correction was applied, and the resulting FDR-adjusted *q*-values were considered significant at *q* < 0.05. Partial correlation analyses adjusting for age, sex, and LEDD were used to examine independent association between *nan_Akk_* levels and Hoehn–Yahr stage. Associations between categorical clinical severity (Hoehn–Yahr stages 1–3 vs. 4–5) and clinical or microbial variables were analyzed using binary multivariable logistic regression, adjusting for age at examination, sex, disease duration, LEDD, body mass index (BMI), and Constipation Severity Score. An additional model further adjusted for antiparkinsonian medication classes (dopamine agonists, monoamine oxidase-B (MAO-B) inhibitors, and catechol-O-methyltransferase (COMT) inhibitors). In addition, to account for the ordinal nature of Hoehn–Yahr staging, ordered logistic regression analysis was performed using Hoehn–Yahr stage as the outcome variable, with *nan_Akk_* levels as the primary variable of interest and age at examination, sex, disease duration, and LEDD as covariates.To determine the diagnostic and discriminative performance of qPCR-based *nanAkk* quantification, receiver operating characteristic (ROC) curve analyses were performed. The area under the curve (AUC) with 95% confidence intervals (CIs) was calculated to assess discriminative ability. Optimal cutoff values were determined by maximizing the Youden index (*J* = sensitivity + specificity − 1), with the corresponding sensitivity and specificity obtained. To account for potential confounding factors, generalized linear model (GLM) analysis was additionally performed for the association between the predicted abundance of the F-type ATPase module and the relative abundance of the genus *Blautia*. The model included age at examination, sex, LEDD, disease duration, Constipation Severity Score, and MDS–UPDRS Part III as covariates.

The sample size was determined based on feasibility and participant availability during the study period, with the aim of capturing sufficient variability to detect group differences. No formal sample size calculation was conducted.

## Results

### Characteristics of individuals and clinical data

This cross-sectional study included 65 healthy controls (mean age: 67.2 years; range: 50–88 years; 53.8% male) and 59 patients with PD (mean age: 69.6 years; range: 39–88 years; 47.5% male). There were no significant differences in age (*P* = 0.2691) or sex distribution (*P* = 0.4773) between the control and PD groups. The demographic and clinical characteristics of the participants are shown in Table 1.

**Table 1.** Clinical characteristics of participants in control and PD groups.

	Control (*N* = 65) mean ± SD (range)	PD (*N* = 59) mean ± SD (range)	*p*-value
Age at examination (years)	67.2 ± 12.7 (50–88)	69.6 ± 10.7 (39–88)	0.2691
Sex (Male/female)	35/30	28/31	0.4773
Disease duration (months)		93.4 ± 65.3 (2–287)	
LEDD (mg)		742 ± 629 (25–3490)	
MDS-UPDRS I		11.7 ± 6.7 (2–34)	
MDS-UPDRS II		17.3 ± 9.3 (1–36)	
MDS-UPDRS III		31.0 ± 16.5 (3–67)	
MDS-UPDRS IV		3.94 ± 4.10 (0–14)	
Hoehn-Yahr stage		2.85 ± 1.05 (1–5)	
MMSE		26.1 ± 3.8 (13–30)	
ACE-R		84.4 ± 13.5 (45–99)	
MoCA-J		20.9 ± 4.8 (10–29)	
FAB		13.4 ± 3.2 (4–18)	
PDQ-39		59.3 ± 28.5 (2–114)	
GDS-15		6.36 ± 3.78 (0–14)	
OSIT-J		4.36 ± 2.89 (0–10)	
SCOPA-AUT		15.8 ± 9.4 (1–50)	
ESS		8.95 ± 5.75 (1–24)	
RBDSQ-J		5.17 ± 3.39 (0–13)	

Significance was tested using the Wilcoxon rank sum test. PD, Parkinson's disease; LEDD, levodopa equivalent daily dose; MDS–UPDRS, Movement Disorder Society–Unified Parkinson's Disease Rating Scale; MMSE, Mini-Mental State Examination; ACE-R, Addenbrooke's Cognitive Examination-Revised; MoCA-J, Japanese version of the Montreal Cognitive Assessment; FAB, Frontal Assessment Battery; PDQ-39, Parkinson's Disease Questionnaire-39; GDS-15, Geriatric Depression Scale-15; OSIT-J, Odor Stick Identification Test for Japanese; SCOPA-AUT, Scales for Outcomes in Parkinson's Disease-Autonomic; ESS, Epworth Sleepiness Scale; RBDSQ-J, Japanese version of the REM Sleep Behavior Disorder Screening Questionnaire.

### Microbial diversity among study groups

To investigate microbiota alterations associated with PD, fecal microbial communities were analyzed using 16S rRNA gene NGS. Supplemental Figure 2 illustrates the species- and genus-level composition of the fecal microbiota across study groups.

**Figure 1.**
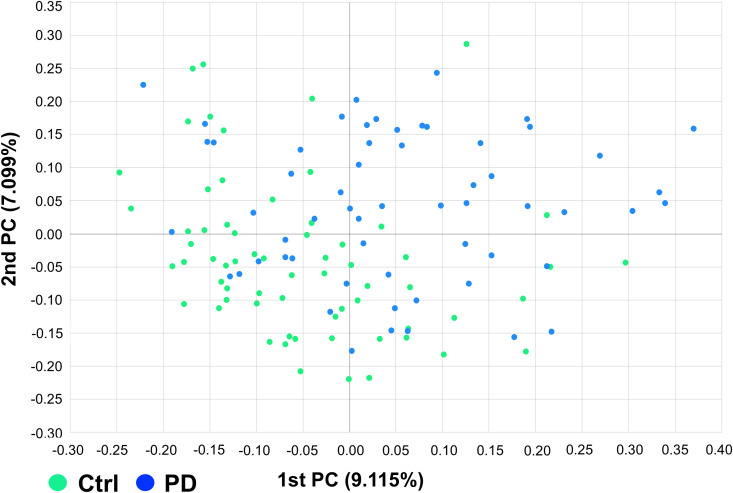
Principal coordinate analysis plots based on generalized UniFrac distances, illustrating differences in fecal microbiota composition between healthy controls and patients with PD. Ctrl, control; PD, Parkinson's disease.

Alpha diversity (measured by the Shannon and Chao1 indices) was significantly higher in the PD group compared with the control group (*P* = 0.026 and *P* = 0.018, respectively). In addition, beta diversity (assessed by principal coordinates analysis based on generalized UniFrac distances) showed distinct separation between the groups, suggesting compositional differences in microbial communities (Fig. 1). These differences were further supported by PERMANOVA, which confirmed statistically significant differences between the PD and control groups (*P* < 0.001).

**Figure 2.**
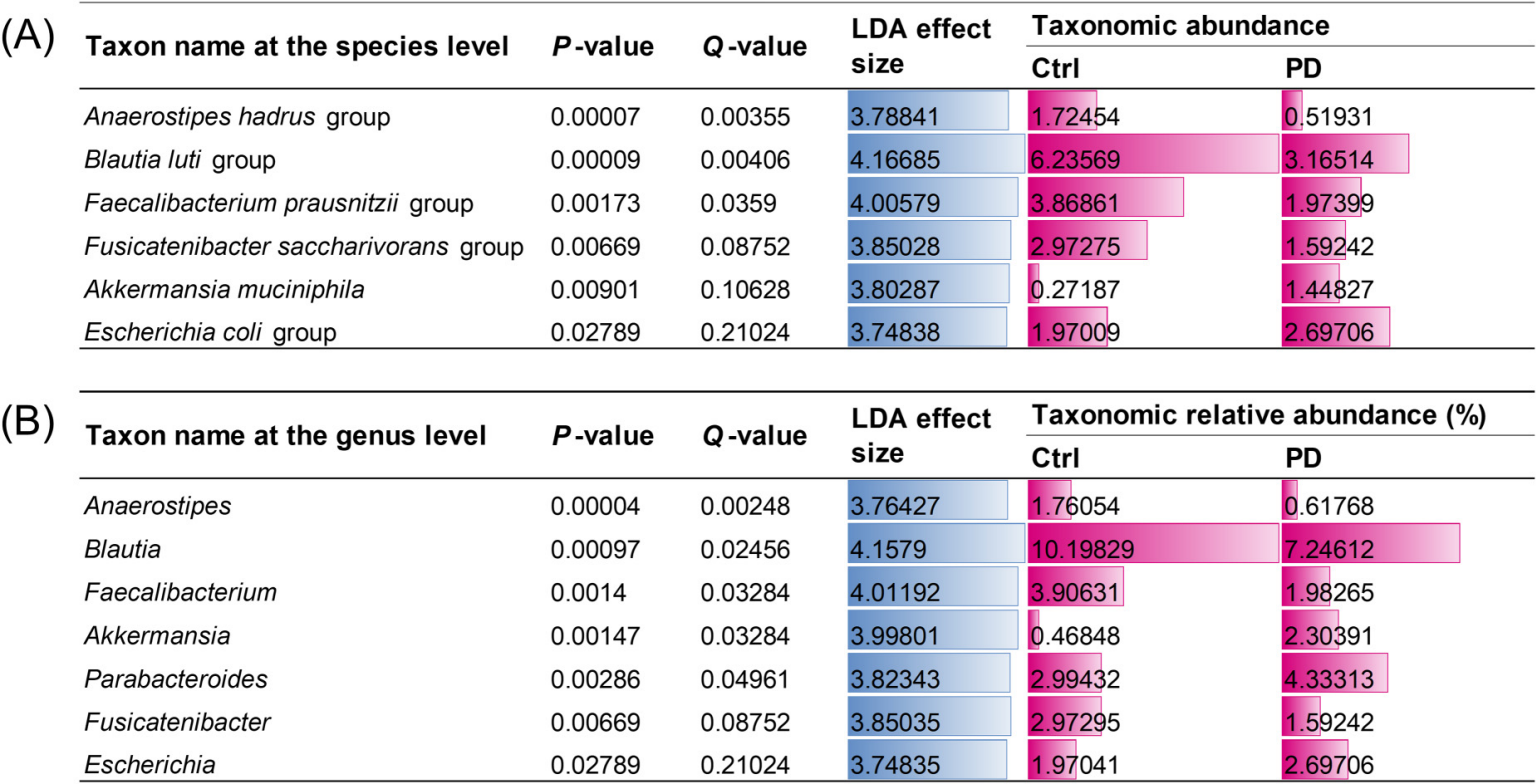
LEfSe results identifying differences in fecal microbiota composition between healthy controls and patients with PD. Differences at the species level (A) and genus level (B) were identified by LEfSe. The analysis used an LDA effect size threshold >3.7. Blue bars represent the magnitude of LDA effect size, while red bars indicate the relative abundance of each taxon. Taxa are listed in ascending order of *P*-values. LEfSe, linear discriminant analysis effect size; LDA, linear discriminant analysis; Ctrl control; PD, Parkinson's disease.

To identify taxa differentially associated with PD, LEfSe analysis was performed. At the species level, four species were significantly less abundant in the PD group: *Anaerostipes hadrus* group, *Blautia luti* group, *Faecalibacterium prausnitzii* group, and *Fusicatenibacter saccharivorans* group, while two species, *A. muciniphila* and *Escherichia coli* groups, were significantly more abundant (Fig. 2A).

At the genus level, corresponding trends were observed: *Anaerostipes*, *Blautia*, *Faecalibacterium*, and *Fusicatenibacter* were significantly less abundant in the PD group, whereas *Akkermansia*, *Escherichia*, and *Parabacteroides* were significantly more abundant (Fig. 2B).

### Correlation between key gut microbes and clinical parameters of Pd

To explore the clinical relevance of gut microbiota alterations in PD, correlations were examined between LEfSe-identified taxa and clinical parameters. Among the taxa identified by LEfSe, *Blautia* showed weak negative correlations with motor severity. At the species level, *Blautia luti* correlated with Hoehn–Yahr stage but did not remain significant after FDR correction. In contrast, at the genus level, *Blautia* abundance showed a similar negative association that remained significant after FDR correction. Another SCFA-producing genus, *Anaerostipes*, also showed a weak negative association with LEDD, although this relationship was not significant after FDR correction.

By comparison, *Akkermansia* demonstrated a moderate positive correlation with both measures of motor severity (Hoehn–Yahr stage and MDS–UPDRS Part III), representing the strongest correlations observed among all taxa assessed in this study. Furthermore, *Akkermansia* was negatively correlated with multiple cognitive measures (MMSE, MoCA-J, ACE-R, and FAB). Among these, the associations with MMSE and MoCA-J remained significant after FDR correction. This indicates a consistent direction of association between higher *Akkermansia* abundance and poorer cognitive performance. These selected results are summarized in Table 2, which lists correlations with *P* < 0.05 and |*r_s_*| > 0.3. Because multiple comparisons were performed, associations that did not remain significant after FDR correction should be interpreted cautiously.

**Table 2.** Selected correlations between key gut microbes and clinical parameters in the PD group.

Taxa	Clinical parameters	*r_s_*	*P-value*	*q-value*
*Blautia luti*	Hoehn–Yahr stage	−0.3014	0.0203	0.3866
*Blautia*	Age at examination	−0.3493	0.0067	0.0635
	Hoehn–Yahr stage	−0.3860	0.0025	0.0481
*Anaerostipes*	LEDD	−0.3202	0.0134	0.2548
*Akkermansia*	Disease duration	0.3028	0.0197	0.0625
	MDS-UPDRS Part III	**0**.**4299**	**0**.**0008**	**0**.**0080**
	Hoehn–Yahr stage	**0**.**4991**	**<0**.**0001**	**0**.**0011**
	MMSE	−0.3427	0.0079	0.0374
	ACE-R	−0.3006	0.0207	0.0562
	MOCA-J	−0.3483	0.0096	0.0435
	FAB	−0.3157	0.0158	0.0599

Correlations were calculated using Spearman's rank-order method with false discovery rate (FDR) correction for multiple comparisons. The FDR correction was applied across the 19 clinical parameters tested in the PD group. The table lists only taxa–clinical parameter pairs that are highlighted and discussed in the text (*P* < 0.05, |*r_s_*| > 0.30). Values with |*r_s_*| > 0.40, *P* < 0.001, and *q* < 0.05 are shown in bold. Associations that did not remain significant after FDR correction are presented for exploratory purposes. PD: Parkinson's disease; LEDD: levodopa equivalent daily dose; MDS–UPDRS: Movement Disorder Society–Unified Parkinson's Disease Rating Scale; MMSE: Mini-Mental State Examination; ACE-R: Addenbrooke's Cognitive Examination Revised; MOCA-J: Japanese version of the Montreal Cognitive Assessment; FAB: Frontal Assessment Battery.

### qPCR-based quantification of *nan_Akk_*

**Figure 3.**
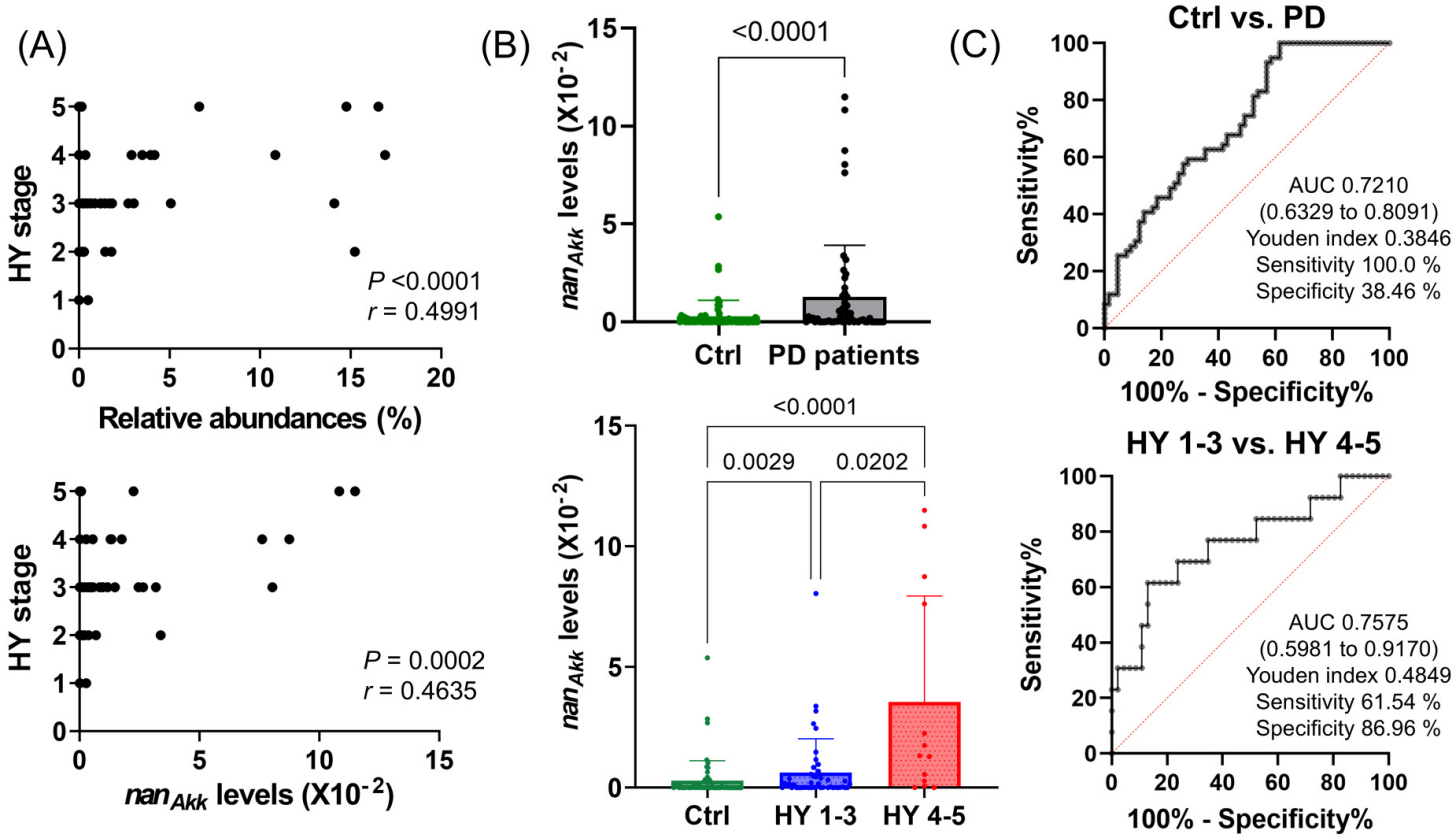
Association between *nan_Akk_* abundance and motor severity in the PD group. (A): Scatter plots showing positive Spearman correlations between the relative abundance of *Akkermansia* (top) or *nan_Akk_* levels (bottom) and motor severity, as assessed by HY stage. (B): The *nan_Akk_* gene levels in PD patients compared with controls (top), and across severity groups (Ctrl, HY stages 1–3, and stages 4–5; bottom). Bars represent the mean with standard deviation shown as positive error bars. *P*-values are shown above each comparison. (C): ROC analysis for the discrimination capacity of *nan_Akk_* levels among groups. ROC curves and AUC for distinguishing between healthy controls and PD (top), and HY stages 1–3 and stages 4–5 in the PD group (bottom). Numbers in brackets indicate 95% confidence intervals for the AUC. *nan_Akk_*, *Akkermansia nan* gene; PD, Parkinson's disease; HY, Hoehn–Yahr; Ctrl, controls; ROC, receiver operating characteristic; AUC, area under the curve.

Since the relative abundance of *Akkermansia* showed the strongest correlation with Hoehn–Yahr stage, we next quantified *nanAkk* gene levels by qPCR as a proxy for its mucin-degrading potential, and determined whether *nanAkk* levels also reflected motor severity. As shown in Fig. 3A, representative scatter plots illustrate positive Spearman correlations between both the relative abundance of *Akkermansia* or *nan_Akk_* levels and Hoehn–Yahr stage (*P* < 0.0001, *r_s_* = 0.4991; *P* = 0.0002, *r_s_* = 0.4635, respectively).

As shown in Fig. 3B (top panel), *nan_Akk_* levels were significantly higher in patients with PD compared with controls (*P* < 0.0001). We then assessed the clinical relevance of *nan_Akk_* gene levels by comparing participants stratified by motor severity (Fig. 3B, bottom panel). This showed that *nan_Akk_* levels were significantly higher in both the Hoehn–Yahr stage 1–3 group and stage 4–5 group compared with the control group (*P* = 0.0029 and *P* < 0.0001, respectively). Moreover, *nan_Akk_* levels in the stage 4–5 group were significantly higher than in the stage 1–3 group (*P* = 0.0202), suggesting a stepwise increase in mucin-degrading *nan_Akk_* abundance with disease severity. These results highlight the potential of *nan_Akk_* levels as a microbiota-derived marker, reflecting not only the presence of PD but also its clinical severity.

Next, ROC analysis demonstrated moderate discriminative ability of *nan_Akk_* quantification for distinguishing patients with PD from healthy controls (AUC = 0.7210) and for differentiating advanced PD (Hoehn–Yahr stages 4–5) from mild PD (stages 1–3) (AUC = 0.7575) (Fig. 3C). To further examine the additive predictive value of *nan_Akk_*, we compared the AUCs of ROC curves between the baseline model (age + sex) and extended model (including *nan_Akk_*) using a nonparametric method for correlated ROC curves implemented in JMP Pro 18. For PD versus control discrimination, adding *nan_Akk_* significantly improved the AUC from 0.5716 to 0.6618 (ΔAUC = 0.0902, *P* = 0.0183). For disease severity classification (Hoehn–Yahr 1–3 vs. 4–5), inclusion of *nan_Akk_* increased the AUC from 0.6622 to 0.7492 (ΔAUC = 0.0870), although this difference did not reach statistical significance (*P* = 0.2336).

To strengthen these observations, a partial correlation analysis was performed while adjusting for potential confounders including age at examination, sex, and LEDD. Correlation between *nan_Akk_* levels and Hoehn–Yahr stage remained statistically significant after adjustment (*P* = 0.0015, *r* = 0.4042).

Finally, binary logistic regression analysis showed that *nan_Akk_* levels remained significantly associated with motor severity (Hoehn–Yahr stages 1–3 vs. 4-5) even after adjustment for age at examination, sex, disease duration, LEDD, BMI, and Constipation Severity Score according to the likelihood ratio test (Supplemental Table 1A). In an additional model adjusted for antiparkinsonian medication classes, the association was significant according to both the Wald test and the likelihood ratio test (Supplemental Table 1B). An ordered logistic regression analysis further showed that higher *nan_Akk_* levels were significantly associated with increasing Hoehn–Yahr stage, independent of covariates (Supplemental Table 2).

In contrast, no significant associations were found between *nan_Akk_* levels and systemic metabolic indices or NLR as an inflammatory marker. There was a weak association with plasma succinic acid, but this did not remain significant after FDR correction (Supplemental Table 3).

### NGS analysis of amplified *nan_Akk_* gene fragments

To validate the specificity of the primer set targeting the *nanAkk* gene, NGS was performed on PCR-amplified fragments initially detected by qPCR analysis. These fragments were obtained from stool samples of the five PD patients with the highest *Akkermansia* abundance in the Hoehn–Yahr stage 4–5 group. Supplemental Table 4 summarizes the characteristics of the major amplicon sequence variants (ASVs). Four dominant ASVs were identified from the total reads, all of which matched the *nan_Akk_* gene sequences of either *A. muciniphila* or *A. massiliensis*, showing almost perfect sequence identity.

### PICRUSt-based prediction of microbial metabolic pathways

Functional predictions using Phylogenetic Investigation of Communities by Reconstruction of Unobserved States (PICRUSt) revealed several differentially abundant microbial metabolic pathways between the PD and control groups. Among these, F-type ATPase (prokaryotes and chloroplasts) (Kyoto Encyclopedia of Genes and Genomes [KEGG] module M00157) exhibited the most statistically significant difference (*P* = 4.11 × 10^−6^), with lower predicted abundance in the PD group (Table 3). Correlation analysis was further performed between the predicted abundances of KEGG modules and the taxa identified by LEfSe. Among these, the F-type ATPase module showed a positive correlation with relative abundance of the genus *Blautia* (*P* < 0.0001, *r_s_* = 0.5026). This relationship was further tested using a GLM including age at examination, sex, LEDD, disease duration, Constipation Severity Score, and MDS–UPDRS Part III as covariates. Relative abundance of the genus *Blautia* was the only variable that remained significant (*P* = 0.0013) (Supplemental Table 5).

**Table 3.** Differentially abundant KEGG functional modules predicted by PICRUSt analysis in healthy controls and patients with PD.

Module	Definition	Orthology	Pathway	*P*-value	*q*-value	LDA effect size	Predicted abundance	
							Ctrl	PD
M00157	F-type ATPase, prokaryotes and chloroplasts	K02111, K02112, K02113, K02114, K02115, K02108, K02109, K02110	ko00190, ko00195	4.115E-06	4.828E-04	2.834	1.440	1.298
M00793	dTDP-L-rhamnose biosynthesis	K00973, K01790	ko00521, ko00523, ko00541, ko01100, ko01110, ko01250	1.390E-04	4.331E-04	2.564	0.770	0.702
M00127	Thiamine biosynthesis, prokaryotes, AIR (+DXP/tyrosine) ≥ TMP/TPP	K03154	ko00730, ko01100, ko01240	6.042E-04	1.051E-02	2.520	1.124	1.060

KEGG modules with significantly different predicted abundances between groups were identified using the LEfSe algorithm. Only modules with *P* < 0.001 are shown. LDA effect size indicates the discriminatory power of each module. Predicted abundance values represent mean relative functional contributions (arbitrary units) of each module in the Ctrl and PD groups, respectively. Correction for multiple comparisons was performed using the false discovery rate across all KEGG modules included in the analysis. KEGG: Kyoto Encyclopedia of Genes and Genomes; PICRUSt: Phylogenetic Investigation of Communities by Reconstruction of Unobserved States; PD: Parkinson's disease; LDA: linear discriminant analysis; Ctrl: control; ATPase: adenosine triphosphatase; dTDP: deoxythymidine diphosphate; AIR: 5-aminoimidazole ribonucleotide; DXP: 1-deoxy-D-xylulose 5-phosphate; TMP: thiamine monophosphate; TPP: thiamine pyrophosphate.

## Discussion

In this cross-sectional study, we characterized gut microbial features linked to both the presence and severity of PD, with a particular focus on the genus *Akkermansia* and its *nan_Akk_* gene, a key component of the sialic acid-catabolizing *nan* gene cluster. While 16S rRNA gene sequencing-based profiling delineated the taxonomic composition of the gut microbiota, qPCR-based quantification of *nan_Akk_* allowed indirect assessment of the metabolic potential associated with mucin degradation, providing a clinically applicable approach to microbiome evaluation.

In the present study, we first identified specific gut microbial taxa associated with PD, largely consistent with previous reports.^2–4^ Notably, four SCFA-related taxa were significantly depleted in the PD group: the *Blautia luti* group, a potential acetate-producing taxon that may support butyrate-producing bacteria through metabolic cross-feeding,^35,36^ and three butyrate-producing taxa—the *Anaerostipes hadrus* group, *Faecalibacterium prausnitzii* group, and *Fusicatenibacter saccharivorans* group.^37,38^ The reduction of these SCFA-producing taxa is biologically plausible, as SCFAs—particularly butyrate—play key roles in maintaining intestinal barrier integrity, regulating immune responses, and exerting anti-inflammatory effects; these mechanisms are increasingly implicated in PD pathophysiology through the gut–brain axis.^37^

In addition, the abundance of *Akkermansia muciniphila* and the *Escherichia coli* group was significantly increased in the PD group. A key finding of this study was the strong association between *Akkermansia* abundance and motor severity. This pattern was paralleled by qPCR-based *nan_Akk_* levels, which progressively increased with Hoehn–Yahr stage. However, previous studies have reported inconsistent findings regarding the relationship between *Akkermansia* and disease severity in PD. For example, a previous large cohort study found no significant association between *Akkermansia* abundance and disease severity.^39^ In contrast, another study suggested that *Akkermansia* may be associated with early disease progression,^4^ while other studies have highlighted its context-dependent role as either beneficial or detrimental.^7^ These discrepancies may reflect differences in cohort characteristics, disease stage, sample size, and analytical approaches. Importantly, most prior studies have focused on taxonomic abundance based on 16S rRNA sequencing, whereas the present study assessed a functional gene marker (*nan_Akk_*) that reflects mucin-degrading metabolic potential. This difference in methodological focus may partly explain why our findings demonstrate an association with disease severity. In this context, qPCR-based quantification of *nan_Akk_* may provide a practical approach to linking microbial composition with functional potential. In addition, qPCR offers a simple, rapid, and low-cost method that can be readily applied in large-scale or longitudinal settings. Accordingly, *nan_Akk_* may represent a candidate marker associated with disease severity, although further validation is required. These findings may also be interpreted in the context of prior literature suggesting that enhanced mucin degradation may thin the mucus layer and alter host–microbe signaling at the epithelial interface, potentially contributing to local immune activation and possibly influencing disease progression.^4,5^

Notably, *nan_Akk_* showed no clear relationship with circulating metabolites or inflammatory indices, suggesting that its clinical relevance may not be driven by systemic metabolic or inflammatory changes reported in previous studies.^5^ Discrepancies between local and systemic measures may stem from complex regulation of host homeostasis, potential confounders such as medications, and temporal mismatch between microbial and physiological dynamics in this cross-sectional study design. Future studies combining stool inflammatory markers, microbial metabolites, and mucosal transcriptomic analyses will clarify how *Akkermansia* activity interfaces with host physiology.

Sequencing of *nan_Akk_* amplicons confirmed that our assay robustly detected both *A. muciniphila* and *A. massiliensis*, demonstrating that it captures *Akkermansia* species relevant to mucin-degrading metabolic potential. Although our present NGS data do not establish comprehensive coverage of all *Akkermansia nan* loci, the detection of multiple species suggests that the assay does not selectively target a single lineage but instead broadly amplifies *nan_Akk_* across the genus. The limited variation in *nan_Akk_* sequences observed by NGS may reflect PCR amplification or library preparation biases. Beyond analytical validation, this qPCR approach offers a practical and scalable alternative to metagenomic profiling for monitoring PD-related microbial changes. The ability to replicate 16S-derived findings through a single gene assay highlights its translational utility.

Functional pathway analysis using PICRUSt showed a reduction in the F-type ATPase module in PD, which was positively correlated with the relative abundance of *Blautia*. However, because this module is widely conserved among gut microbes and PICRUSt only infers function from 16S rRNA data, the apparent reduction may partly reflect annotation bias, compositional shifts, or limitations of prediction. Thus, this finding may indicate altered microbial metabolic potential in PD; however, its biological significance remains uncertain and should be interpreted cautiously. Therefore, this finding should be regarded as exploratory and hypothesis-generating rather than conclusive.

This study has several limitations that should be acknowledged. First, the observed associations between gut microbial features and disease severity are correlational, because our cross-sectional design does not allow causal inference or assessment of predictive value for disease progression. In addition, the ROC analyses showed only moderate discriminatory performance. Future longitudinal or prospective studies will be required to validate these findings. Second, although increased *nan_Akk_* abundance suggests enhanced potential for sialic acid utilization, this does not directly indicate ongoing mucin degradation or gut barrier impairment. In this context, the mechanistic pathways linking *Akkermansia* abundance to PD severity should therefore be considered hypotheses based on prior literature rather than conclusions derived from the present study. Additional markers of mucosal integrity, including measurements of mucus layer integrity, gut permeability, and local mucosal inflammatory responses, as well as markers of enteric or central nervous system involvement, are needed to clarify the functional consequences. Third, the relatively modest sample size and single-center recruitment may limit the generalizability of our findings. Independent replication in larger and more diverse cohorts will be necessary. Fourth, obesity- and diet-related metadata were incomplete in our cohort. Although BMI was available and considered in multivariable analyses within the PD group, corresponding obesity- and dietary/nutritional information was not available in the control group. Therefore, residual confounding related to body composition or nutritional factors cannot be excluded in the case–control comparisons. Fifth, although PICRUSt-based functional predictions and qPCR quantification of *nan_Akk_* provide valuable functional insights, these approaches do not directly measure gene expression or enzymatic activity. Future studies integrating metatranscriptomics, proteomics, or metabolomic analyses will be important to validate and extend the biological relevance of our findings.

In conclusion, *nan_Akk_* serves as a non-invasive microbial gene marker reflecting mucin-degrading metabolic potential, which in turn is linked to both the presence and severity of PD. While 16S rRNA-based profiling primarily delineates the taxonomic structure of the gut microbiota, qPCR-based *nan_Akk_* quantification provides insight into microbial functional potential by targeting a gene marker associated with mucin degradation and offers a rapid and cost-effective approach. This framework of microbial functional potential may provide a basis for future biomarker development and patient stratification, and warrants further investigation in prospective studies.

## Supplemental Material

sj-docx-1-pkn-10.1177_1877718X261462342 - Supplemental material for A qPCR-based approach targeting the microbial gene marker *nanA* of mucin-degrading *Akkermansia* in Parkinson's disease Supplemental material, sj-docx-1-pkn-10.1177_1877718X261462342 for A qPCR-based approach targeting the microbial gene marker *nanA* of mucin-degrading *Akkermansia* in Parkinson's disease by Yasuaki Mizutani, Tadashi Fujii, Yasuhiro Maeda, Kohei Funasaka, Eizaburo Ohno, Yoshiki Hirooka, Hirohisa Watanabe and Takumi Tochio in Journal of Parkinson's Disease

sj-docx-3-pkn-10.1177_1877718X261462342 - Supplemental material for A qPCR-based approach targeting the microbial gene marker *nanA* of mucin-degrading *Akkermansia* in Parkinson's disease Supplemental material, sj-docx-3-pkn-10.1177_1877718X261462342 for A qPCR-based approach targeting the microbial gene marker *nanA* of mucin-degrading *Akkermansia* in Parkinson's disease by Yasuaki Mizutani, Tadashi Fujii, Yasuhiro Maeda, Kohei Funasaka, Eizaburo Ohno, Yoshiki Hirooka, Hirohisa Watanabe and Takumi Tochio in Journal of Parkinson's Disease

sj-docx-4-pkn-10.1177_1877718X261462342 - Supplemental material for A qPCR-based approach targeting the microbial gene marker *nanA* of mucin-degrading *Akkermansia* in Parkinson's disease Supplemental material, sj-docx-4-pkn-10.1177_1877718X261462342 for A qPCR-based approach targeting the microbial gene marker *nanA* of mucin-degrading *Akkermansia* in Parkinson's disease by Yasuaki Mizutani, Tadashi Fujii, Yasuhiro Maeda, Kohei Funasaka, Eizaburo Ohno, Yoshiki Hirooka, Hirohisa Watanabe and Takumi Tochio in Journal of Parkinson's Disease

sj-docx-5-pkn-10.1177_1877718X261462342 - Supplemental material for A qPCR-based approach targeting the microbial gene marker *nanA* of mucin-degrading *Akkermansia* in Parkinson's disease Supplemental material, sj-docx-5-pkn-10.1177_1877718X261462342 for A qPCR-based approach targeting the microbial gene marker *nanA* of mucin-degrading *Akkermansia* in Parkinson's disease by Yasuaki Mizutani, Tadashi Fujii, Yasuhiro Maeda, Kohei Funasaka, Eizaburo Ohno, Yoshiki Hirooka, Hirohisa Watanabe and Takumi Tochio in Journal of Parkinson's Disease

sj-xlsx-6-pkn-10.1177_1877718X261462342 - Supplemental material for A qPCR-based approach targeting the microbial gene marker *nanA* of mucin-degrading *Akkermansia* in Parkinson's disease Supplemental material, sj-xlsx-6-pkn-10.1177_1877718X261462342 for A qPCR-based approach targeting the microbial gene marker *nanA* of mucin-degrading *Akkermansia* in Parkinson's disease by Yasuaki Mizutani, Tadashi Fujii, Yasuhiro Maeda, Kohei Funasaka, Eizaburo Ohno, Yoshiki Hirooka, Hirohisa Watanabe and Takumi Tochio in Journal of Parkinson's Disease

sj-docx-7-pkn-10.1177_1877718X261462342 - Supplemental material for A qPCR-based approach targeting the microbial gene marker *nanA* of mucin-degrading *Akkermansia* in Parkinson's disease Supplemental material, sj-docx-7-pkn-10.1177_1877718X261462342 for A qPCR-based approach targeting the microbial gene marker *nanA* of mucin-degrading *Akkermansia* in Parkinson's disease by Yasuaki Mizutani, Tadashi Fujii, Yasuhiro Maeda, Kohei Funasaka, Eizaburo Ohno, Yoshiki Hirooka, Hirohisa Watanabe and Takumi Tochio in Journal of Parkinson's Disease

sj-xlsx-8-pkn-10.1177_1877718X261462342 - Supplemental material for A qPCR-based approach targeting the microbial gene marker *nanA* of mucin-degrading *Akkermansia* in Parkinson's disease Supplemental material, sj-xlsx-8-pkn-10.1177_1877718X261462342 for A qPCR-based approach targeting the microbial gene marker *nanA* of mucin-degrading *Akkermansia* in Parkinson's disease by Yasuaki Mizutani, Tadashi Fujii, Yasuhiro Maeda, Kohei Funasaka, Eizaburo Ohno, Yoshiki Hirooka, Hirohisa Watanabe and Takumi Tochio in Journal of Parkinson's Disease

sj-docx-9-pkn-10.1177_1877718X261462342 - Supplemental material for A qPCR-based approach targeting the microbial gene marker *nanA* of mucin-degrading *Akkermansia* in Parkinson's disease Supplemental material, sj-docx-9-pkn-10.1177_1877718X261462342 for A qPCR-based approach targeting the microbial gene marker *nanA* of mucin-degrading *Akkermansia* in Parkinson's disease by Yasuaki Mizutani, Tadashi Fujii, Yasuhiro Maeda, Kohei Funasaka, Eizaburo Ohno, Yoshiki Hirooka, Hirohisa Watanabe and Takumi Tochio in Journal of Parkinson's Disease

sj-docx-10-pkn-10.1177_1877718X261462342 - Supplemental material for A qPCR-based approach targeting the microbial gene marker *nanA* of mucin-degrading *Akkermansia* in Parkinson's disease Supplemental material, sj-docx-10-pkn-10.1177_1877718X261462342 for A qPCR-based approach targeting the microbial gene marker *nanA* of mucin-degrading *Akkermansia* in Parkinson's disease by Yasuaki Mizutani, Tadashi Fujii, Yasuhiro Maeda, Kohei Funasaka, Eizaburo Ohno, Yoshiki Hirooka, Hirohisa Watanabe and Takumi Tochio in Journal of Parkinson's Disease

sj-docx-11-pkn-10.1177_1877718X261462342 - Supplemental material for A qPCR-based approach targeting the microbial gene marker *nanA* of mucin-degrading *Akkermansia* in Parkinson's disease Supplemental material, sj-docx-11-pkn-10.1177_1877718X261462342 for A qPCR-based approach targeting the microbial gene marker *nanA* of mucin-degrading *Akkermansia* in Parkinson's disease by Yasuaki Mizutani, Tadashi Fujii, Yasuhiro Maeda, Kohei Funasaka, Eizaburo Ohno, Yoshiki Hirooka, Hirohisa Watanabe and Takumi Tochio in Journal of Parkinson's Disease
