# Supplementary material for: A qPCR-based approach targeting the microbial gene marker nanA of mucin-degrading Akkermansia in Parkinson's disease
Source: J Parkinsons Dis. 2026 Aug 10:1877718X261462342. Online ahead of print. doi: 10.1177/1877718X261462342 (PMC13461883; doi:10.1177/1877718X261462342)
Supplement: Supplementary file 1 [file sj-docx-1-pkn-10.1177_1877718X261462342.docx]

**Supplemental Figure 1.** Nucleotide sequence alignment of *Akkermansia* sp. *nanA* gene homologs from *Akkermansia* strains. The red lettered sequence above the alignment indicates the region selected for primer design. Nucleotide sequence alignment was performed using Color Align Conservation (https://www.bioinformatics.org/sms2/color_align_cons.html).

Accession numbers and corresponding strains:
CP001071.1: Akkermansia muciniphila ATCC BAA-835

PJLM01000002.1: Akkermansia muciniphila strain BSM09

DNOI01000233.1: Akkermansia muciniphila isolate UBA10767

PJKN01000005.1: Akkermansia muciniphila strain GP43

JABFCI010000002.1: Akkermansia sp. GGCC_0220

CP156916.1: Akkermansia massiliensis strain Akk273

PJKB01000001.1: Akkermansia muciniphila strain GP22

JABDHQ010000025.1: Akkermansia muciniphila strain CSUN-56

VVMV01000002.1: Akkermansia sp. BIOML-A60

SUVM01000028.1: Akkermansia bacterium isolate SIG72

**Supplemental Figure 2.** Fecal microbiota composition in healthy controls and patients with PD. Mean relative abundances of taxonomic groups are shown at the genus level (A) and species level (B). PD, Parkinson’s disease; Ctrl, control.
