# Supplementary material for: A qPCR-based approach targeting the microbial gene marker nanA of mucin-degrading Akkermansia in Parkinson's disease
Source: J Parkinsons Dis. 2026 Aug 10:1877718X261462342. Online ahead of print. doi: 10.1177/1877718X261462342 (PMC13461883; doi:10.1177/1877718X261462342)
Supplement: Supplementary file 2 [file sj-docx-10-pkn-10.1177_1877718X261462342.docx]

**Supplemental Methods 2**

**Measurement of purine nucleotides and related cofactors in whole blood**

**Sample collection and processing**

After a fasting period of more than 6 hours, venous blood was collected into EDTA 2Na-containing tubes. A 500 μL of whole blood was mixed with an equal volume of ice-cold 8% perchloric acid (PCA), vortexed immediately, and centrifuged at 12,000 × g for 5 min at 4 °C. The resulting supernatant was stored at −150 °C until analysis.

**Instruments**

A Nexera UHPLC system (Shimadzu, Kyoto, japan) was used, consisting of a gradient pump, a vacuum degasser and auto sampler. An Intrada Organic Acid column (150 x 2 mm, Imtakt, Kyoto, Japan) was used at 60 °C. A 5 μL of analyte solution was injected to the column and eluted at flow 0.2 ml/min using step gradient alternating between A; a mixture of acetonitrile/100 mM ammonium acetate = 95:5 and B; a mixture of acetonitrile/100 mM ammonium acetate = 10:90. The gradient began with 15% B, and was then programmed as follows: 0–5 min, gradient to 55% B; 5–5.1 min, gradient to 70% B; 5.1–10 min, gradient to 100% B; 10–10.1 min, gradient back to 15% B; 10.1–13 min, hold at 15% B to re-equilibrate the column.

An LCMS-8060 or LCMS-8060NX triple quadrupole mass spectrometer (Shimadzu) equipped with an electrospray ionization source was used for MS/MS analysis.

**Internal standard solution**

A mixed internal standard (IS) solution of ATP-d_4_, ADP-^15^N_5_, AMP-^13^C_5_, NAD-d_4_, and NMN-d_4_ was prepared to by diluting the respective stock solution to 10 μM.

**Standard mixture**

A mixture of ATP, ADP, AMP, NAD, NADH, and NMN was prepared to by diluting the respective stock solution to 200 μM. The mixture was diluted to make 100, 50, 25, 12, 6, 3, 1.5, 0.75, 0.4, 0.2, 0.1μM of each solution.

**Sample preparation**

A mixture of 90 μL of 1% formic acid in acetonitrile, 10 μL of IS solution and 20 μL of whole blood treated by perchloric acid or standard mixture were added to a well of OSTRO sample preparation plate (Waters, Milford, MA, USA). The mixture was mixed by pipetting up and down. After mixing, place the OSTRO plate and collection plate on a positive pressure processor and set the flow for 60 psi for 5 min. The elution in the collection plate was analyzed by LC-MS/MS.

**Quantified analytes**

Final quantitative analyses were performed for AMP, ADP, ATP, NAD, NADP, and NMN. NADH and NADPH were below the detection limit following PCA treatment and were therefore excluded from subsequent analyses.
