# Supplementary material for: A qPCR-based approach targeting the microbial gene marker nanA of mucin-degrading Akkermansia in Parkinson's disease
Source: J Parkinsons Dis. 2026 Aug 10:1877718X261462342. Online ahead of print. doi: 10.1177/1877718X261462342 (PMC13461883; doi:10.1177/1877718X261462342)
Supplement: Supplementary file 3 [file sj-docx-11-pkn-10.1177_1877718X261462342.docx]

**Supplemental Methods 3**

**Measurement of energy metabolism–associated plasma metabolites**

**Sample collection and processing**

After a fasting period of more than 6 hours, venous blood was collected into EDTA 2Na-containing tubes. To obtain plasma, samples were centrifuged at 1,500 × *g* for 10 minutes, and 500 μL aliquots of plasma supernatant were transferred into polypropylene tubes and stored at −80 °C until analysis.

**UHPLC–MS/MS analysis of plasma metabolites**

An internal standard mixture in EtOH was prepared as a mixture of 150 μM acetic acid d4, 80 μM succinic acid d4, 100 μM caproic acid d3, 100 μM caprylic acid d3 and 100 μM lauric acid d3. To 15 μL plasma sample in 75% methanol solution (30 μL) was added the internal standard mixture (15 μL), 7.5% pyridine solution in 75% methanol (30 μL), 50 mM 3-nitrophenylhydrazine solution in 75% methanol (30 μL) and 50 mM 1-ethyl-3-(3-dimethylaminopropyl) carbodiimide solution in 75% methanol (30 μL). After the mixture was shaken at room temperature for 30 min, 0.5% formic acid solution in 75% methanol (600 μL) was added. The mixture was centrifuged (15, 000× g for 10 min at 4°C) then the supernatant was injected to UHPLC-MS/MS (LCMS-8060NX; Shimadzu, Kyoto, Japan). Analytes were eluted from a reverse phase column (Mastro 2 C18, 150 x 2 mm, 3 μm; Shimadzu GLC, Tokyo, Japan) with the gradient method using a mobile phase with 0.1% formic acid in water and acetonitrile at a flow rate of 0.35 mL/min, were detected by multiple reaction monitoring mode of MS/MS.

**Analyzed metabolites**

Several organic acids, including propionic acid, butyric acid, isobutyric acid, valeric acid, isovaleric acid, malonic acid, isocitric acid, oxaloacetic acid, caprylic acid, capric acid, and caproic acid, were below the detection sensitivity of this method and could not be reliably quantified. Final analyses included one short-chain fatty acid (acetic acid), one medium-chain fatty acid (lauric acid), and 11 TCA-related intermediates (lactic acid, pyruvic acid, acetoacetic acid, 3-hydroxybutyric acid, 2-hydroxybutyric acid, 2-oxobutyric acid, citric acid, 2-ketoglutaric acid, succinic acid, fumaric acid, and malic acid). The lactic acid/pyruvic acid ratio was also examined.
