# Supplementary material for: A qPCR-based approach targeting the microbial gene marker nanA of mucin-degrading Akkermansia in Parkinson's disease
Source: J Parkinsons Dis. 2026 Aug 10:1877718X261462342. Online ahead of print. doi: 10.1177/1877718X261462342 (PMC13461883; doi:10.1177/1877718X261462342)
Supplement: Supplementary file 4 [file sj-docx-3-pkn-10.1177_1877718X261462342.docx]

**Supplemental Tables 1A and 1B.** Binary multivariable logistic regression including *nan_Akk_* levels and other potential confounders associated with Hoehn-Yahr stage (1-3 vs 4-5) in the PD group.

**Table 1A.**

| **Covariate/Factor** | Estimate | Wald χ^2^ | *P*-value (Wald) | 95% CI Lower | 95% CI Upper | LR χ^2^ | *P*-value (LR) |
| --- | --- | --- | --- | --- | --- | --- | --- |
| ***nan_Akk_* levels** | 6.320×10^-1^ | 2.164 | 0.1413 | 7.025×10^-2^ | 1.729 | 5.478 | **0.0193** |
| **Age at examination (years)** | 1.276×10^-1^ | 3.220 | 0.0727 | 9.113×10^-3^ | 2.970×10^-1^ | 4.578 | **0.0324** |
| **Sex (0 = Male; 1 = Female)** | -1.027 | 0.921 | 0.3372 | -3.349 | 1.016 | 0.964 | 0.3261 |
| **Disease duration (months)** | 4.750×10^-3^ | 0.230 | 0.6313 | -1.605×10^-2^ | 2.546×10^-2^ | 0.229 | 0.6324 |
| **LEDD (mg)** | 1.089×10^-3^ | 1.638 | 0.2005 | -5.038×10^-4^ | 2.958×10^-3^ | 1.777 | 0.1825 |
| **BMI** | -5.786×10^-1^ | 6.599 | **0.0102** | -1.138 | -2.262×10^-1^ | 13.957 | **0.0002** |
| **Constipation Severity Score** | -4.830×10^-1^ | 2.429 | 0.1191 | -1.222 | 5.499×10^-2^ | 3.040 | 0.0812 |

**Table 1B.**

| **Covariate/Factor** | Estimate | Wald χ^2^ | *P*-value (Wald) | 95% CI Lower | 95% CI Upper | LR χ^2^ | *P*-value (LR) |
| --- | --- | --- | --- | --- | --- | --- | --- |
| ***nan_Akk_* levels** | 1.554 | 4.0725 | **0.0436** | 4.418×10^-1^ | ∞ | 11.437 | **0.0007** |
| **Age at examination (years)** | 2.094×10^-1^ | 3.4492 | 0.0633 | 4.169×10^-2^ | 3.953×10^-1^ | 7.109 | **0.0077** |
| **Sex (0 = Male; 1 = Female)** | 1.533 | 0.3930 | 0.5307 | -2.510 | **6.845** | 0.457 | 0.4989 |
| **Disease duration (months)** | -1.288×10^-2^ | 0.5324 | 0.4656 | -4.970×10^-2^ | 2.380×10^-2^ | 0.534 | 0.4648 |
| **LEDD (mg)** | 1.767×10^-3^ | 2.1295 | 0.1445 | -2.566×10^-4^ | 4.767×10^-3^ | 2.860 | 0.0908 |
| **BMI** | -7.100×10^-1^ | 5.8225 | **0.0158** | -1.246 | -2.805×10^-1^ | 14.943 | **0.0001** |
| **Constipation Severity Score** | -4.056×10^-1^ | 1.2683 | 0.2601 | -1.278 | 2.427×10^-1^ | 1.460 | 0.2270 |
| **Dopamine agonist use**  **(0 = No; 1 = Yes)** | 4.951 | 2.4280 | 0.1192 | 2.373×10^-1^ | 9.612 | 4.340 | **0.0372** |
| **MAO-B inhibitor use**  **(0 = No; 1 = Yes)** | 2.037 | 1.0818 | 0.2983 | -1.508 | 6.379 | 1.215 | 0.2704 |
| **COMT inhibitor use**  **(0 = No; 1 = Yes)** | -3.859 | 2.3822 | 0.1227 | -8.289 | -2.947×10^-1^ | 4.730 | **0.0296** |

A binary multivariable logistic regression model was constructed with Hoehn–Yahr stage dichotomized as 1–3 (mild–moderate) versus 4–5 (advanced) as the dependent variable. The model included *nan_Akk_* levels as the primary variable of interest. The following potential confounders were included as covariates: age at examination, sex, disease duration, LEDD, BMI, and Constipation Severity Score. The Constipation Severity Score was derived by summing items 5 and 6 of the SCOPA-AUT and was treated as a continuous measure of constipation severity. Supplementary Table 1A presents the base multivariable model including these clinical covariates. Supplementary Table 1B presents an additional model further adjusted for antiparkinsonian medication classes, including the use of dopamine agonists, COMT inhibitors, and MAO-B inhibitors (coded as binary variables: 0 = no, 1 = yes). 95% CIs were derived from the Wald statistics. LR χ^2^ and *P*-value (LR) were used to evaluate improvement in overall model fit when each variable was included. *nan_Akk_*, *Akkermansia nan* gene; PD, Parkinson's disease; CI, confidence interval； LR, likelihood ratio; LEDD, levodopa equivalent daily dose; BMI, body mass index; MAO-B, monoamine oxidase B; COMT, Catechol-O-methyltransferase; SCOPA-AUT, Scales for Outcomes in Parkinson’s Disease-Autonomic. *Bold letters indicate a correlation with a statistically significant difference.
