# Supplementary material for: A qPCR-based approach targeting the microbial gene marker nanA of mucin-degrading Akkermansia in Parkinson's disease
Source: J Parkinsons Dis. 2026 Aug 10:1877718X261462342. Online ahead of print. doi: 10.1177/1877718X261462342 (PMC13461883; doi:10.1177/1877718X261462342)
Supplement: Supplementary file 5 [file sj-docx-4-pkn-10.1177_1877718X261462342.docx]

**Supplemental Table X.** Ordered logistic regression analysis with Hoehn–Yahr stage as the outcome in the PD group.

| **Covariate/Factor** | Estimate | Wald χ^2^ | *P*-value (Wald) | OR | 95% CI Lower | 95% CI Upper | LR χ^2^ | *P*-value (LR) |
| --- | --- | --- | --- | --- | --- | --- | --- | --- |
| ***nan_Akk_* levels** | 3.529×10^-1^ | 9.052 | **0.0026** | 1.4232 | 1.1309 | 1.7911 | 11.666 | **0.0006** |
| **Age at examination (years)** | 1.023×10^-1^ | 12.772 | **0.0004** | 1.1077 | 1.0473 | 1.1717 | 14.908 | **0.0001** |
| **Sex (0 = Male; 1 = Female)** | -6.024×10^-1^ | 1.301 | 0.2540 | 0.5475 | 0.1944 | 1.5414 | 1.221 | 0.2691 |
| **Disease duration (months)** | 2.917×10^-3^ | 0.392 | 0.5314 | 1.0029 | 0.9938 | 1.0121 | 0.431 | 0.5113 |
| **LEDD (mg)** | 8.443×10^-4^ | 3.038 | 0.0814 | 1.0008 | 0.9999 | 1.0018 | 1.810 | 0.1785 |

Hoehn–Yahr stage was treated as an ordinal outcome variable. The model included *nan_Akk_* levels as the primary variable of interest and age at examination, sex, disease duration, and LEDD as covariates. ORs and 95% CIs were calculated from the estimated regression coefficients. *nan_Akk_*, *Akkermansia nan* gene; PD, Parkinson’s disease; OR, odds ratio; CI, confidence interval; LEDD, levodopa equivalent daily dose. *Bold letters indicate a correlation with a statistically significant difference.
