# Supplementary material for: A qPCR-based approach targeting the microbial gene marker nanA of mucin-degrading Akkermansia in Parkinson's disease
Source: J Parkinsons Dis. 2026 Aug 10:1877718X261462342. Online ahead of print. doi: 10.1177/1877718X261462342 (PMC13461883; doi:10.1177/1877718X261462342)
Supplement: Supplementary file 6 [file sj-docx-5-pkn-10.1177_1877718X261462342.docx]

**Supplemental Table 3.** Associations of *nan*_Akk_ levels with systemic metabolic or inflammatory indices in the PD group.

| **Biomarker indices** | ***rs*** | ***P-value*** | ***q-value*** |
| --- | --- | --- | --- |
| Whole blood AMP (μM) | -0.0480 | 0.7382 | 1.0000 |
| Whole blood ADP (μM) | 0.0073 | 0.9593 | 1.0000 |
| Whole blood ATP (μM) | 0.0447 | 0.7554 | 1.0000 |
| Whole blood NAD (μM) | 0.1149 | 0.4220 | 1.0000 |
| Whole blood NADP (μM) | -0.1518 | 0.2877 | 1.0000 |
| Whole blood NMN (μM) | -0.0099 | 0.9449 | 1.0000 |
| Plasma lactic acid (μM) | -0.2361 | 0.0988 | 0.6915 |
| Plasma pyruvic acid (μM) | -0.2482 | 0.0822 | 0.8631 |
| Plasma lactate / pyruvate ratio | 0.1135 | 0.4327 | 1.0000 |
| Plasma acetoacetic acid (μM) | -0.0182 | 0.9002 | 1.0000 |
| Plasma 3-hydroxybutyric acid (μM) | 0.0257 | 0.8594 | 1.0000 |
| Plasma 2-hydroxybutyric acid (μM) | -0.0350 | 0.8093 | 1.0000 |
| Plasma 2-oxobutyric acid (μM) | -0.0619 | 0.6694 | 1.0000 |
| Plasma citric acid (μM) | -0.0764 | 0.5980 | 1.0000 |
| Plasma 2-ketoglutaric acid (μM) | 0.0043 | 0.9765 | 0.9765 |
| Plasma succinic acid (μM) | 0.2850 | **0.0449** | 0.9419 |
| Plasma fumaric acid (μM) | 0.1379 | 0.3397 | 1.0000 |
| Plasma malic acid (μM) | 0.0189 | 0.8965 | 1.0000 |
| Plasma acetic acid (μM) | 0.0429 | 0.7675 | 1.0000 |
| Plasma lauric acid (μM) | -0.1056 | 0.4749 | 1.0000 |
| NLR | 0.1363 | 0.3403 | 1.0000 |

Spearman’s rank correlation test was used to determine significant correlations, followed by correction for multiple comparisons by FDR. The FDR correction was applied across the 21 systemic metabolic or inflammatory indices tested. Bold letters indicate a correlation with a statistically significant difference. PD, Parkinson’s disease; AMP, adenosine monophosphate; ADP, adenosine diphosphate; ATP, adenosine triphosphate; NAD, nicotinamide adenine dinucleotide; NADP, nicotinamide adenine dinucleotide phosphate; NMN, nicotinamide mononucleotide; NLR, neutrophil-to-lymphocyte ratio; FDR, false discovery rate. Statistical significance was defined as p < 0.05 and q < 0.05 (FDR-adjusted).
