# Supplementary material for: A qPCR-based approach targeting the microbial gene marker nanA of mucin-degrading Akkermansia in Parkinson's disease
Source: J Parkinsons Dis. 2026 Aug 10:1877718X261462342. Online ahead of print. doi: 10.1177/1877718X261462342 (PMC13461883; doi:10.1177/1877718X261462342)
Supplement: Supplementary file 7 [file sj-docx-7-pkn-10.1177_1877718X261462342.docx]

**Supplemental Table 5.** The results of the generalized linear models for the F-type ATPase module based on the relative abundance of genus *Blautia* and potential confounders in the PD group.

| **Covariate/Factor** | Estimate | LR chi-square | *P*-value | 95% CI Lower | 95% CI Upper |
| --- | --- | --- | --- | --- | --- |
| **Relative abundance of genus *Blautia*** | 1.414 × 10^-2^ | 10.33 | **0.0013** | 5.762 × 10^-3^ | 2.251 × 10^-2^ |
| **Age at examination (years)** | -1.154 × 10^-3^ | 0.4981 | 0.4803 | -4.406 × 10^-3^ | 2.098 × 10^-3^ |
| **Sex (0 = Male; 1 = Female)** | 1.807 × 10^-2^ | 0.3191 | 0.5721 | -4.561 × 10^-2^ | 8.175 × 10^-2^ |
| **LEDD (mg)** | -1.404 × 10^-5^ | 0.2131 | 0.6443 | -7.463 × 10^-5^ | 4.654 × 10^-5^ |
| **Disease duration (months)** | -6.419 × 10^-5^ | 0.0546 | 0.8153 | -6.119 × 10^-4^ | 4.835 × 10^-4^ |
| **Constipation Severity Score** | -3.116 × 10^-3^ | 0.1499 | 0.6986 | -1.915 × 10^-2^ | 1.291 × 10^-2^ |
| **MDS-UPDRS III** | -5.791 × 10^-4^ | 0.3430 | 0.5581 | -2.547 × 10^-3^ | 1.389 × 10^-3^ |

The Constipation Severity Score was derived by summing items 5 and 6 of the SCOPA-AUT and was treated as a continuous measure of constipation severity. PD, Parkinson's disease; LR, likelihood ratio; LEDD, levodopa equivalent daily dose; MDS-UPDRS, Movement Disorder Society’s Unified Parkinson's Disease Rating Scale; SCOPA-AUT, Scales for Outcomes in Parkinson's Disease-Autonomic. *Bold letters indicate a correlation with a statistically significant difference.
