# Supplementary material for: A qPCR-based approach targeting the microbial gene marker nanA of mucin-degrading Akkermansia in Parkinson's disease
Source: J Parkinsons Dis. 2026 Aug 10:1877718X261462342. Online ahead of print. doi: 10.1177/1877718X261462342 (PMC13461883; doi:10.1177/1877718X261462342)
Supplement: Supplementary file 8 [file sj-docx-9-pkn-10.1177_1877718X261462342.docx]

**Supplemental Methods 1**

**16S rRNA gene next-generation sequencing of fecal DNA samples**

The 16S rRNA gene NGS of fecal DNA samples was performed following a previously described protocol.^1^ Briefly, DNA was extracted from fecal samples using the QIAamp PowerFecal Pro DNA Kit (QIAGEN, Venlo, The Netherlands), following the manufacturer’s instructions. A primer set comprising Pro341F and Pro805R targeting the V3–V4 region of the bacterial 16S rRNA gene was used for PCR.^2^ The 16S rRNA gene NGS was conducted at the Techno Suruga Laboratory (Shizuoka). Paired-end sequencing (2 × 300 bp) was performed using the MiSeq platform (Illumina, San Diego, CA, USA), as described previously.^3^ The data output from the MiSeq sequencing system was processed, statistically analyzed, and graphed using the EzBioCloud 16S database and 16S microbiome pipeline provided by ChunLab Inc. (EzBioCloud 16S-based MTP app, available at [https://www.EZbiocloud.net](https://www.ezbiocloud.net/)).^4^ Chao1 estimation and Shannon diversity index were used to assess the richness and evenness of microbial samples, representing alpha diversity. Beta diversity, which reflects the overall phylogenetic distances among groups, was estimated using the Generalized UniFrac distance and visualized via principal coordinate analysis (PCoA). Differences in alpha and beta diversity between the groups were tested using the Wilcoxon rank-sum test and permutational multivariate analysis of variance (PERMANOVA) with 9999 permutations, respectively. Potential biomarkers distinguishing each group were identified using the linear discriminant analysis (LDA) effect size (LEfSe) algorithm, following the default parameters. The analysis used thresholds of *P* < 0.05 and |LDA effect size| > 3.7. Functional metagenomic profiles were inferred using the PICRUSt-based approach integrated in the EZBioCloud pipeline, which predicts functional gene content from 16S rRNA data. Differences in Kyoto Encyclopedia of Genes and Genomes (KEGG) modules between groups were subsequently identified using LEfSe with default settings.

**Primer design**

To develop a primer set capable of collectively quantifying the *nanA* gene encoding N-acetylneuraminate lyase (NanA) in the genus *Akkermansia*, we first identified homologous protein sequences using BLASTp. The query sequence was the NanA protein from *Akkermansia muciniphila* ATCC BAA-835 (GenBank accession no. ACD05759.1), and the search was conducted via the NCBI website (accessed on July 18, 2025). A total of ten homologous amino acid sequences belonging to *Akkermansia* spp. were retrieved. These sequences were annotated as N-acetylneuraminate lyase and were consistently located adjacent to genes encoding N-acylglucosamine 2-epimerase (*nanE*) within the same genomic regions, indicating conserved operon structures across the genus. The corresponding nucleotide sequences were downloaded and aligned using MAFFT (available at GenomeNet; accessed on July 18, 2025). Conserved regions among the *Akkermansia nanA* homologs identified through multiple sequence alignment (as shown in Supplemental Figure 1) served as the basis for designing a genus-specific primer set targeting the *nanA* gene. The primers were designed to amplify a region approximately spanning positions 421–440 bp (forward) and 580–597 bp (reverse) of the *nanA* gene from *A. muciniphila* ATCC BAA-835. The sequences of the primers are as follows:

- Forward primer (Akk_nanA_F): 5′‑taygayatycccgtgctgac‑3′
- Reverse primer (Akk_nanA_R): 5′‑ccaytcgtccacdcccca‑3′

**Quantitative PCR Analysis**

Quantitative PCR (qPCR) was performed to determine the abundances of the *Akkermansia nanA gene* (*nan_Akk_*) and the total bacterial 16S rRNA genes using the QuantStudio 3 Real-Time PCR System (Thermo Fisher Scientific, Waltham, MA, USA). Reaction mixtures were prepared with PowerTrack SYBR Green Master Mix (Thermo Fisher Scientific) according to the manufacturer’s protocol. Gene-specific primer sets for *nan_Akk_* were Akk_nanA_F and Akk_nanA_R, as described above. For total bacterial 16S rRNA gene quantification, F_Bact 1369 and R_Prok 1492 primers were used, as previously described.^2^ The qPCR thermal cycling protocol included an initial denaturation step at 95 °C for 2 min, followed by 40 cycles of denaturation at 95 °C for 10 s, annealing at gene-specific temperatures (55 °C for *nan_Akk_* and 60 °C for 16S rRNA) for 15 s, and extension at 72 °C for 15 s. A final extension step at 72 °C for 1 min was included in all reactions. The *nan_Akk_* levels were calculated by normalizing its copy number to that of the total 16S rRNA gene, allowing for consistent quantification across samples. Absolute quantification of *nan_Akk_* was achieved using PCR-amplified fragments of the *nan_Akk_* gene derived from the *Akkermansia muciniphila* NBRC 114322 genome.

**NGS analysis of amplified *nan_Akk_* gene fragments**

NGS was performed on *nan_Akk_* gene fragments that were initially identified by qPCR from the five patients with the highest *Akkermansia* abundance in the Hoehn–Yahr stage 4–5 group. The amplified fragments were purified using the Wizard SV Gel and PCR Clean-Up System (Promega, Madison, WI, USA) and eluted in 30 µL of the provided elution buffer. The purified DNA served as a template for a secondary PCR to incorporate Illumina adapter sequences. The forward primer used was Akk_nanA_forNGS_F (5′‑ACACTCTTTCCCTACACGACGCTCTTCCGATCTtaygayatycccgtgctgac‑3′), and the reverse primer was Akk_nanA_forNGS_R (5′‑GTGACTGGAGTTCAGACGTGTGCTCTTCCGATCTccaytcgtccacdcccca‑3′). Lowercase letters in each primer correspond to the Akk_nanA_F and Akk_nanA_R primer sequences, while the 5′ flanking sequences represent Illumina adapter overhangs. The secondary PCR was conducted using Quick Taq HS DyeMix (Toyobo, Osaka, Japan) with a final primer concentration of 3.2 pM. The cycling conditions consisted of an initial denaturation at 94 °C for 2 min, followed by one cycle of 55 °C for 15 s and 72 °C for 15 s, then 22 cycles of denaturation at 95 °C for 5 s, annealing at 68 °C for 15 s, and extension at 72 °C for 15 s. A final extension step at 72 °C for 1 min was performed. Following amplification confirmation, PCR products were purified again using the Wizard SV Gel and PCR Clean-Up System and eluted in 50 µL of elution buffer. The resulting amplicons were subjected to paired-end sequencing (2 × 300 bp) on the Illumina MiSeq platform, conducted by Bioengineering Lab Co., Ltd. (Kanagawa, Japan).

1.	Fujii T, Kuzuya T, Kondo N, et al. Altered intestinal Streptococcus anginosus and 5α-reductase gene levels in patients with hepatocellular carcinoma and elevated Bacteroides stercoris in atezolizumab/bevacizumab non-responders. *J Med Microbiol*; 73. Epub ahead of print September 2024. DOI: 10.1099/jmm.0.001878.

2.	Takahashi S, Tomita J, Nishioka K, et al. Development of a prokaryotic universal primer for simultaneous analysis of Bacteria and Archaea using next-generation sequencing. *PLoS One* 2014; 9: e105592.

3.	Fukuda K, Takahashi S, Okutome T. Clinical relevance of sequencing faecal Microbiota in ulcerative colitis patients, consanguineous and non-consanguineous relatives. *Research Square*. Epub ahead of print June 22, 2022. DOI: 10.21203/rs.3.rs-1640143/v1.

4.	Yoon S-H, Ha S-M, Kwon S, et al. Introducing EzBioCloud: a taxonomically united database of 16S rRNA gene sequences and whole-genome assemblies. *Int J Syst Evol Microbiol* 2017; 67: 1613–1617.
